# Editorial: Single cell dynamics and cell cycle length variation

**DOI:** 10.3389/fcell.2023.1321316

**Published:** 2023-10-24

**Authors:** Dirk Loeffler

**Affiliations:** ^1^ Department of Hematology, St. Jude Children’s Research Hospital, Memphis, TN, United States; ^2^ Department of Pathology and Laboratory Medicine, The University of Tennessee, Memphis, TN, United States

**Keywords:** cell cycle, cell division, cell fate decisions, cilia, transcription factor condensates, single-cell dynamics, hematopoietic stem cells, pluripotent stem cells

How cells make decisions is one of the great mysteries of life. Understanding the molecular mechanisms regulating cell fate decisions is key to manipulating cell behavior to produce safe cell therapy products used for gene editing and transplants. Cell fate decisions, including proliferation, apoptosis, differentiation, self-renewal, and migration are regulated by signals from the microenvironment and the cellular state at the time when signals are received. Both environmental signals and cellular states are dynamic and change constantly creating heterogenous cellular responses of otherwise homogenous cell populations (Haas et al., 2018). Changes in cell cycle phases are among the best-known examples contributing to different cellular states and heterogeneity. However, the relationship between cell cycle, cell fate decisions, heterogeneity, and other dynamic processes in single cells, including signaling, metabolism, and their role in differentiation remain poorly understood. In this Research Topic, an excellent review and three original research articles present recent advances in our understanding of how the cell cycle regulates cell fate decisions.

Treichel and Filippi review the role of the cell cycle in hematopoietic stem cell (HSC) fate decisions and discuss evidence that HSC fate and cell cycle progression are coupled. HSCs are mostly quiescent and only divide occasionally to generate progeny when needed. After division HSC daughter cells can either retain stem cell properties and return to dormancy and/or continue to divide to differentiate into mature blood cells. However, not all HSCs are the same and their function can be stratified based on cell cycle kinetics and their divisional history. As HSC function declines progressively with an increasing number of past divisions, HSC fate decisions seem to be inherently linked to the cell cycle (Bernitz et al., 2016). However, the precise nature of this relationship is unknown. Here, Treichel and Filippi discuss and summarize recent insights that shed light on this relationship and highlight evidence that provides important clues for future research:

1) HSC exit from quiescence is regulated by the CDK6/cyclin D complex as CDK6^High^ HSCs enter the cell cycle faster than CDK6^Low^ HSCs (Laurenti et al., 2015), 2) HSC daughter cells after asymmetric division differ in metabolic activity and cell cycle kinetics (Loeffler et al., 2019; Loeffler et al., 2022), and 3) loss of the trithorax protein ASH2l leads to deregulation of mitosis associated genes, inhibits proliferation, differentiation and induces epigenetic changes (Lüscher-Firzlaff et al., 2019).

The original research provided in this Research Topic further explores the relationship between cell cycle and cell fate determination. Work in pluripotent stem cells (PSCs) identified the G1 phase of the cell cycle as the critical “window of opportunity” when cells are sensitive to signals that induce changes in gene expression required for differentiation. PSCs in G1 respond to differentiation cues whereas cells in S or G2 phases do not respond until the following cell cycle (Pauklin and Vallier, 2013). During differentiation, PSCs slowly downregulate the core pluripotency transcription factors OCT4, SOX2, and NANOG, but the onset of differentiation can be detected earlier based on the nuclear redistribution of these factors (Verneri et al., 2020). Following these reports, Oses et al. focused on the cell cycle-dependent localization and redistribution of OCT4, SOX2, and NANOG during PSC differentiation. They hypothesized that the S-phase functions as a window of opportunity to execute changes in chromatin in cells that received differentiation cues in G1. Using live cell imaging of fluorescent reporter for OCT4, SOX2, and HP1α the authors quantified how OCT4 and SOX2 condensates redistribute during cell cycle progression. They specifically focused on transcription factor redistribution in S-phase using the cell cycle reporter PCNA that can distinguish early-, mid-, and late S-phase. Oses et al. discovered that in response to differentiation signals in G1 pluripotency transcription factors redistribute during the early-S to mid-S transition. Although further work is required, this work suggests that the early S-phase is a critical time window when chromatin remodels to execute fate decisions.

Jiang et al. investigated how the Fibroblast Growth Factor Receptor 1 Oncogene Partner (FOP) regulates cell cycle progression. FOP is a centrosomal protein involved in microtubule anchoring, ciliogenesis, and cancer development. Primary cilia are antenna-like organelles formed by microtubules, which are present in quiescent cells (G0 phase) and are resorbed when cells re-enter the cell cycle (Pugacheva et al., 2007). Primary cilia must be completely disassembled before mitosis to release the centrioles required for mitotic spindle formation. Using immunofluorescence imaging of fixed cell lines, Jiang et al. show that knockdown of FOP increases cilia length, while ectopic overexpression of FOP suppresses cilia growth. FOP thus is a negative regulator of ciliogenesis and promotes cell cycle re-entry of quiescent cells by facilitating cilia disassembly.

Didaskalou et al. interrogate the role of the Hepatoma Upregulated Protein (HURP) during division. During division, proper mitotic spindle assembly and function are critical for faithful chromosome segregation into the two daughter cells and therefore cell cycle progression. The mitotic spindle is responsible for the accurate segregation of chromosomes and consists of microtubules, motor proteins, and microtubule-associated proteins, including HURP, a spindle-assembly factor that bundles and stabilizes kinetochore fibers (Koffa et al., 2006). HURP is essential for proper chromosome segregation, but its spatiotemporal dynamics and localization during division are poorly understood. Using Photoactivation and Fluorescence Recovery After Photobleaching of GFP-tagged HURP in HeLA cells, Didaskalou et al. identify several novel HURP interaction partners, show HURP interacts with distinct complexes in metaphase, and demonstrate that its spatiotemporal dynamics depend on phosphorylation at Ser627.

Understanding the mechanisms underlying fate decisions will improve our understanding of stem cell function and differentiation. This knowledge will allow us to manipulate cell fate decisions to produce high-quality cell therapy products at scale. Recent research presented in this Research Topic and elsewhere suggests that cell fate decisions are intrinsically linked to the cell cycle and events during cell division (Pauklin and Vallier, 2013; Loeffler et al., 2019; Loeffler et al., 2022). Despite recent progress, our understanding of how the cell cycle regulates cell fate decisions is incomplete, and further research is needed to accomplish the long-sought goal of controlling cell fate decisions.
